# Discovery of potential protein biomarkers associated with sugarcane white leaf disease susceptibility using a comparative proteomic approach

**DOI:** 10.7717/peerj.12740

**Published:** 2022-01-05

**Authors:** Kantinan Leetanasaksakul, Sittiruk Roytrakul, Narumon Phaonakrop, Suthathip Kittisenachai, Siriwan Thaisakun, Nitiya Srithuanok, Klanarong Sriroth, Laurent Soulard

**Affiliations:** 1 Functional Proteomics Technology, National Center for Genetic Engineering and Biotechnology, National Science and Technology Development Agency, Khlong Nueng, Khlong Luang, Pathum Thani, Thailand; 2 Mitr Phol Innovation and Research Center, Khoksa-at, Phu Khiao, Chaiyaphum, Thailand

**Keywords:** Sugarcane white leaf disease, Phytoplasma, Proteomics, Candidate protein biomarkers

## Abstract

Sugarcane white leaf disease (SCWLD) is caused by phytoplasma, a serious sugarcane phytoplasma pathogen, which causes significant decreases in crop yield and sugar quality. The identification of proteins involved in the defense mechanism against SCWLD phytoplasma may help towards the development of varieties resistant to SCWLD. We investigated the proteomes of four sugarcane varieties with different levels of susceptibility to SCWLD phytoplasma infection, namely K88-92 and K95-84 (high), KK3 (moderate), and UT1 (low) by quantitative label-free nano-liquid chromatography-tandem mass spectrometry (nano LC-MS/MS). A total of 248 proteins were identified and compared among the four sugarcane varieties. Two potential candidate protein biomarkers for reduced susceptibility to SCWLD phytoplasma were identified as proteins detected only in UT1. The functions of these proteins are associated with protein folding, metal ion binding, and oxidoreductase. The candidate biomarkers could be useful for further study of the sugarcane defense mechanism against SCWLD phytoplasma, and in molecular and conventional breeding strategies for variety improvement.

## Introduction

Sugarcane (*Saccharum* spp.) is an important economic crop in tropical and subtropical areas. It is regarded as a major source of crystal sugar and the second largest resource for biofuel (Wongkaew, 2012). Sugarcane cultivation is negatively impacted by infectious diseases, and in Thailand, the second-largest sugarcane exporter in the world, sugarcane white leaf disease (SCWLD) is a major problem. SCWLD-affected plants present leaves with chlorosis and proliferating tillers, stunted growth, and un-millable canes (Marcone, 2002). In 2011/12, more than 27,200 hectares of sugarcane plantation in Thailand were affected by SCWLD, resulting in economic losses to the sugar industries of approximately one billion Thai baht (Wongkaew et al., 1997; Office of The Cane and Sugar Board & Ministry of Industry Thailand, OCSB; Wongkaew, 2012). SCWLD is caused by infection with phytoplasma phloem pathogens, which are nonculturable, wall-less prokaryotes residing in the sieve tube elements of the plant (Marcone, 2002). In Thailand, SCWLD phytoplasmas are naturally transmitted by the leafhopper *Matsumuratettix hyroglyphicus* (Matsumoto, Lee & Teng, 1969) and *Yamatotettix flavovittatus* (Hanboonsong et al., 2002).

Since phytoplasma resides in the plant’s phloem systems, it is difficult to eliminate by chemical treatment in commercial fields. Alternatively, breeding of resistant varieties may offer a more practical and efficient approach to mitigate SCWLD compared with conventional thermotherapy and chemical control of insect vectors of phytoplasma (Rao, Mall & Marcone, 2012). Genetic engineering of sugarcane has been applied for crop protection to generate SCWLD-resistant cultivars (Lakshmanan et al., 2005). However, the complexity of the sugarcane genome (Hayes et al., 2021) makes selective genetic modification too time-consuming and impractical (Scortecci et al., 2012). Hence, a greater understanding of the molecular basis for natural variation in SCWLD susceptibility among existing varieties could be applied for the purpose of sugarcane breeding of resistant varieties.

Diverse protein functions have been annotated for plant growth and development, metabolism, and tolerance to pathogenic attack (Sugio & Hogenhout, 2012; Margaria, Abbà & Palmano, 2013; Luge et al., 2014). Proteomic profiling of protein expressions that change in response to infection in plant diseases can give insights into plant defense responses to pathogen invasion (Luge et al., 2014; Li et al., 2019). A proteomic-based comparison of sugarcane varieties with different susceptibilities to sugarcane smut disease provided insights into the interactions between the fungal pathogen and sugarcane plant (Singh et al., 2019). For bacterial pathogens of sugarcane, proteomic profiling has been conducted for sugarcane infected with *Gluconacetobacter diazotrophicus* (Lery et al., 2011).

Label-free quantitative mass spectrometry is a common method for proteomic analysis (Neilson et al., 2011). This technique is employed to quantify protein abundance by combining data from spectral counting and peptide ion intensities of the protein across several samples (Zhu, Smith & Huang, 2010). Previous proteomic studies based on protein separation by one-dimensional gradient polyacrylamide gels (1DE) and two-dimensional gels followed by tandem mass spectrometry (MS) systems were conducted to profile protein expressions in sugarcane under various abiotic and biotic stresses (Jangpromma et al., 2010; Barnabas et al., 2015; Salvato et al., 2019). This technique has also been used to investigate the response to phytoplasma infection in paulownia (Cao et al., 2017) and other plants (Dermastia, Kube & Šeruga Musić, 2019). However, to our knowledge, LC-MS has not been used to study the proteins expressed in sugarcane infected with phytoplasma.

In the present study, we applied the label-free quantification proteomic approach for comparing four sugarcane varieties with differing susceptibilities to phytoplasma infection. We identified proteins expressed only in varieties with low and/or moderate susceptibility to phytoplasma, but not in highly susceptible varieties. These proteins could serve as potential biomarkers in sugarcane breeding programs for selecting phytoplasma-resistant varieties.

## Materials & Methods

### Plant material

The experiments were conducted with four commercial sugarcane cultivars varying in susceptibility to phytoplasma infection: high (K88-92 and K95-84), moderate (KK3) and low (UT1) susceptibility. According to a study in 2019 at the Mitr Phol Innovation and Research center (L Soulard, 2019, unpublished data), K88-92 and K95-84 were identified as significantly more susceptible to SCWLD, while UT1 was characterized as significantly less susceptible to SCWLD. The variety KhonKaen 3 (KK3) was used as a reference since it is the most cultivated variety in Thailand (Khumla et al., 2021).

### Greenhouse experiment

The experiment was a 4  × 4  × 3 design with four genotypes (K88-92, K95-84, UT1 and KK3); four timepoints: *t* = 2 days after inoculation (dai), *t* = 7 dai, *t* = 14 dai and *t* = 30 dai; and three biological replicates (3 individual pots) per variety-timepoint. Timepoints were chosen according to the multiplication and distribution of phytoplasma in infected cane described by Roddee, Kobori & Hanboonsong (2018).

### Inoculation process

The inoculation of plants was performed at two months after planting by insect vector *Matsumuratettix hiroglyphicus* leafhoppers that were previously inoculated with *Candidatus phytoplasma* through consumption of white leaf sugarcane for a minimum of one week. The inoculation treatment consisted of exposing the whole plant to five insect vectors under acrylic cylinders until the target timepoint was reached. Samples were immediately frozen in liquid nitrogen and stored at−80 °C until further sample processing. The leaf samples at 30 dai were used for proteomic analysis.

### Validation of the inoculation by nested PCR

The inoculation of the plants was confirmed by nested PCR after inoculation of all plants. In order to give enough time to the pathogen to multiply and spread systematically in the infected cane (Roddee, Kobori & Hanboonsong, 2018), the nested PCR test was performed at 90 days after inoculation on all plants of the experiment.The test was performed using two sets of primers designed to amplify the 16S-23S rRNA Intergenic Spacer Region of phytoplasma (Hanboonsong et al., 2002). The first set consisted of primers MLO-X (5′- GTTAGGTTAAGTCCTAAAACGAGC-3′) and MLO-Y (5′-GTGCCAAGGCATCCACTGTATGCC-3′) which amplified a 700 bp DNA fragment with the following PCR conditions: 95 °C for 5 min followed by 35 cycles of 95 °C for 1min, 60 °C for 1min and 72 °C for 1 min 30 s, and a final extension at 72 °C for 10 min. Resulting PCR products were diluted 1:40 with sterile water and 1 µl of diluted product was used for the nested PCR performed with the second set of primers. This pair consisted of the primers P1 (5′-GTCGTAACAAGGTATCCCTACCGG-3′) and P2 (5 ′-GGTGGGCCTAAATGGACTTGAACC-3′), which amplified a 210 bp DNA fragment with the following PCR conditions: 95 °C for 2 min followed by 35 cycles of 95 °C for 1 min, 68 °C for 30 s, and 72 °C for 30 s, and a final extension at 72 °C for 10 min. All PCR products were checked by electrophoresis and visualized under UV light using the gel doc XR+ Imager (BioRad, California, USA).

### Protein extraction

To extract the total proteins, sugarcane leaf was pulverized to fine powder in liquid nitrogen. A total of 200 mg of each sample was dissolved by 0.5% sodium dodecyl sulfate (SDS) with continuous vortexing for 30 min at room temperature. The pellets were precipitated by centrifugation at 10,000 g for 5 min. The supernatant was transferred to a new microcentrifuge tube, and subsequently mixed with 0.15% 2,5-dimethoxy-4-chloroamphetamine (DOC) and 72% trichloroacetic acid. The sample was incubated at −20 °C overnight to allow for thorough precipitation. The pellets were collected by centrifugation at 16,000 g for 10 min. The pellet was washed thrice with cold acetone and centrifuged at 16,000 g for 5 min. The pellets were reconstituted in 0.5% SDS, and the protein concentration was determined by the Lowry method (Lowry et al., 1951).

### Protein digestion

Dithiothreitol (DTT) prepared in 10 mM ammonium bicarbonate was added to a final concentration of 10 mM to each 5 µg protein sample to reduce disulfide bonds. The reformation of disulfide bond was prevented by the addition of 30 mM iodoacetamide (IAA) in 10 mM ammonium bicarbonate. The protein samples were digested with 50 ng of sequencing grade porcine trypsin 1:20 (w/w) (Promega, Walldorf, Germany) for 16 h at 37 °C. The peptides were dried under speed vacuum concentrator and resuspended in 0.1% formic acid (FA) prior to nano LC-MS/MS analysis.

### Protein identification and quantification

The extracted peptides were analyzed with the HCTUltra LC-MS system (Bruker Daltonics Ltd; Hamburg, Germany) coupled with a nanoLC system (UltiMate 3000 LC System, Thermo Fisher Scientific; Madison, WI, USA) equipped with an electrospray. Briefly, five microliters of peptide smples separated with the flow rate of 300 nL/min on nanocolumn (Acclaim PepMap™ 100 C18 column 50 mm internal diameter 0.075 mm). Solvent A and B containing 0.1% formic acid in water and 80% acetonitrile, respectively, were used to elute peptides using a linear gradient of 4–70% of solvent B (0–20 min) followed by 90% B from 20–25 min retention time to remove all peptides in the column. Mass spectra (MS) and MS/MS spectra were obtained in the positive-ion mode over the range (m/z) 400–1500 (Compass 1.9 software, Bruker Daltonics).

### Bioinformatics and data analysis

Protein intensities of the LC-MS data were measured based on peptide MS signal intensities by the DeCyder MS2.0 analysis software (GE Healthcare, Chicago, IL, USA). The PepDetect module was performed to evaluate the peptide by producing ion peptides with the following settings as previously described in (Aroonluk, Roytrakul & Jantasuriyarat, 2019): mass resolution, 0.6; typical peak width, 0.1; ion trap mass resolution, 10,000; charge status, from 1 to 4; and m/z shift tolerance, 0.1 u. The PepMatch module evaluated the signal intensity maps from each sample. All MS/MS data from the Decyder MS analysis were completed by applying the global variable mode of carbamidomethyl, variable mode of oxidation (M), peptide charge state (1+, 2+ and 3+), and m/*z* tolerance 0.1 u. These spectra were searched against NCBI protein databases (http://www.ncbi.nlm.nih.gov/) with *Saccharum officinarum* (51,209 sequences; September 2020) to identify matching peptides by using the Mascot software search engine tool 2.3.0 (Matrix Science, London, UK). One gram of BSA was used as an internal standard to normalize protein intensities from each dataset. The mass spectrometry proteomics data have been deposited as PXD028041 for ProteomeXchange and JPST001297 for JPOST. The identified proteins were analyzed with the MultiExperiment Viewer software (MeV, version 4.9.0) and filtered with a one-way ANOVA (*p* < 0.05) (Saeed et al., 2003). Uniport (http://www.uniprot.org/) and search tools were used to identify gene ontology (GO). Venn diagrams were constructed to identify intersections of proteins detected among different sugarcane varieties (Bardou et al., 2014).

## Results

### Detection of SCWLD phytoplasma in sugarcane

The leaf samples collected from inoculation at 90 day post inoculation on the pots were subjected to total DNA extraction and nested PCR analysis. The percentage of individual infection with phytoplasma varied from 41.7% in UT1, 66.7% in K88-92 and K95-84 and 83.3% in KK3 (Table 1). These data confirm upublished reports that UT1 is markedly less susceptible to SCWLD phytoplasma infection than K88-92 and K95-84.

**Table 1:** Percentage of white leaf disease infection of SCWL phytoplasma after 90 day post-inoculation.

Sugarcane varieties	No. infected plants/ no. of test plants	% White leaf disease infection
K88-92	8/12	66.7
K95-84	8/12	66.7
KK3	10/12	83.3
UT1	5/12	41.7

### Proteomic profiling of sugarcane

The shotgun proteomic approach was employed to obtain protein profiles of *Saccharum officinarum* varieties infected with phytoplasma. 248 proteins were identified in total, of which 237, 166, 105, and 129 proteins were detected in the K88-92, K95-84, KK3, and UT1 varieties, respectively.

### Gene ontology analysis

**Figure 1:**
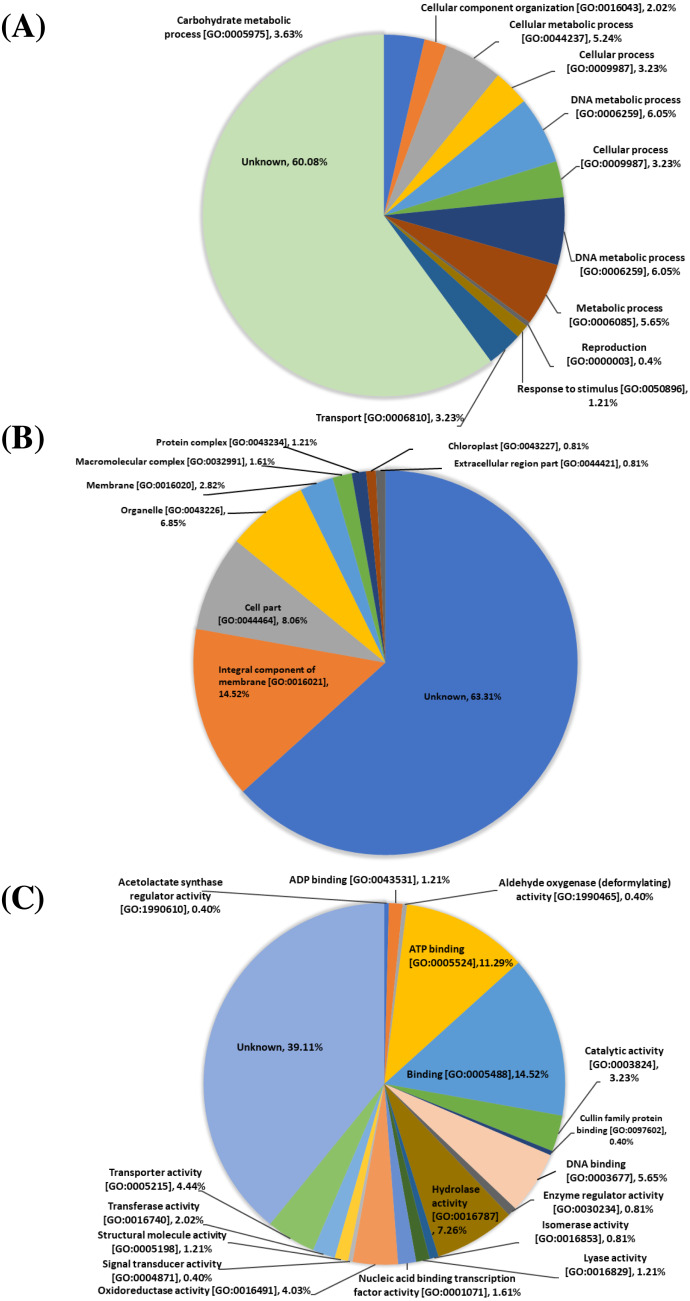
Gene ontology (GO) analysis. Biological functions among the 248 proteins identified from proteomic analysis of four varieties of sugarcane. Pie charts show the represented GO terms at the levels of biological process (A), cellular components (B) and molecular function (C).

The 248 proteins identified from the proteomic data were classified based on gene ontology (GO) functional annotation levels (biological processes, cellular components, and molecular functions). At the biological process level, protein functions include: unknown function (149 proteins, 60.08%), DNA metabolic process [GO:0006259] (30 proteins, 12.10%), cellular process [GO:0009987] (16 proteins, 6.45%), metabolic process (14 proteins, 5.65%), cellular metabolic process [GO:0044237] (13 proteins, 5.24%), carbohydrate metabolic process [GO:0005975] (nine proteins, 3.63%), transport [GO:0006810] (eight proteins, 3.23%), cellular component organization [GO:0016043] (five proteins, 2.02%), response to stimulus [GO:0050896] (three proteins, 1.21%) and reproduction [GO:0000003] (one proteins, 0.40%) (Fig. 1A). At the cellular components level, protein functions include unknown function (157 proteins, 63.31%), integral component of membrane [GO:0016021] (36 proteins, 14.52%), cell part [GO:0044464] (20 proteins, 8.06%), organelle [GO:0043226] (19 proteins, 6.85%) membrane [GO:0016020] (7 proteins, 2.82%), macromolecular complex [GO:0032991] (four proteins, 1.61%), protein complex [GO:0043234] (three proteins, 1.21%), chloroplast [GO:0043227] (two proteins, 0.81%) and extracellular region part [GO:0044421] (two proteins, 0.81%) (Fig. 1B). At the molecular functions level, protein functions include unknown function (97 proteins, 39.11%), binding [GO:0005488] (36 proteins, 14.52%), ATP binding [GO:0005524] (28 proteins, 11.29%), hydrolase activity [GO:0016787] (18 proteins, 7.26%), DNA binding [GO:0003677] (14 proteins, 5.65%), transporter activity [GO:0005215] (11 proteins, 4.44%), oxidoreductase activity (10 proteins, 4.03%), catalytic activity [GO:0003824] (eight proteins, 3.23%), transferase activity [GO:0016740] (five proteins, 2.02%), nucleic acid binding transcription factor activity [GO:0001071] (four proteins, 1.61%), ADP binding [GO:0043531] (three proteins, 1.21%), lyase activity [GO:0016829] (three proteins, 1.21%), structural molecule activity [GO:0005198] (three proteins, 1.21%), enzyme regulator activity [GO:0030234] (two proteins, 0.81%), isomerase activity [GO:0016853] (two proteins, 0.81%), acetolactate synthase regulator activity [GO:1990610] (one protein, 0.40%), aldehyde oxygenase (deformylating) activity [GO:1990465] (one protein, 0.40%), cullin family protein binding [GO:0097602] (one protein, 0.40%) and signal transducer activity [GO:0004871] (one protein, 0.40%) (Fig. 1C)

### Identification of candidate biomarker proteins expressed in sugarcane cultivars with low and moderate susceptibility to phytophora infection

Comparison of protein profiles among the different varieties revealed 53 proteins common to all four varieties, whereas 2, 1 and 36 proteins were identified as uniquely expressed in UT1, KK3 and K88-92, respectively (Fig. 1). We investigated the protein in more detail, in particular the proteins expressed in cultivars with moderate susceptibility to phytoplasma infection (UT1) but not in cultivars with susceptibility (K88-92, K95-84 and KK3) (Table 2). Peptidyl-prolyl cis-trans isomerase and 9-cis-epoxycarotenoid dioxygenase were found to be uniquely expressed in UT1. From these results, we thus identified two proteins that could serve as candidate biomarkers for susceptibility to phytoplasma infection.

Protein ID score: The number reflects the combined scores of all observed mass spectra that can be matched to amino acid sequences within that protein. A higher score indicates a more confident match. Log2 Abundance: The log2 report ion intensities for each spectrum belonging to this protein.

## Discussion

The distribution pattern of SCWL phytoplasma in the sugarcane was reported in the previous study (Roddee, Kobori & Hanboonsong, 2018). This research indicates that SCWL phytoplasma sequentially spreads from the inoculated leaf to all parts of plant, before dispersal to cover the above mature leaves within 35 days of the inoculation. After 90 days of inoculation, SCWL phytoplasma fully distributed in infected young or immature sugarcane plants. For this reason, we investigated potential candidate proteins at 30 days and determined white leaf disease infection of SCWL phytoplasmaat 90 days.

**Table 2:** List of two proteins uniquely expressed in strains with low and moderate susceptibility to white leaf disease.

**Varieties**	**Protein IDs**	**Protein names**	**Protein ID score**	**Peptides**	**Function**	**Log2 Abundance**			
						**K88** **-** **92**	**K95** **-** **84**	**KK3**	**UT1**
**UT1**	A0A059Q1N9	Peptidyl-prolyl cis-trans isomerase	5.98	IVMELYANEVPK	1. Protein folding	0.00	0.00	0.00	19.64
	I6WA52	9-cis-epoxycarotenoid dioxygenase	18.24	VRINLR	1. Metal ion binding 2. Oxidoreductase	0.00	0.00	0.00	16.10

**Notes.**

Protein ID score: The number reflects the combined scores of all observed mass spectra that can be matched to amino acid sequences within that protein. A higher score indicates a more confident match. Log2 Abundance: The log2 report ion intensities for each spectrum belonging to this protein.

**Figure 2:**
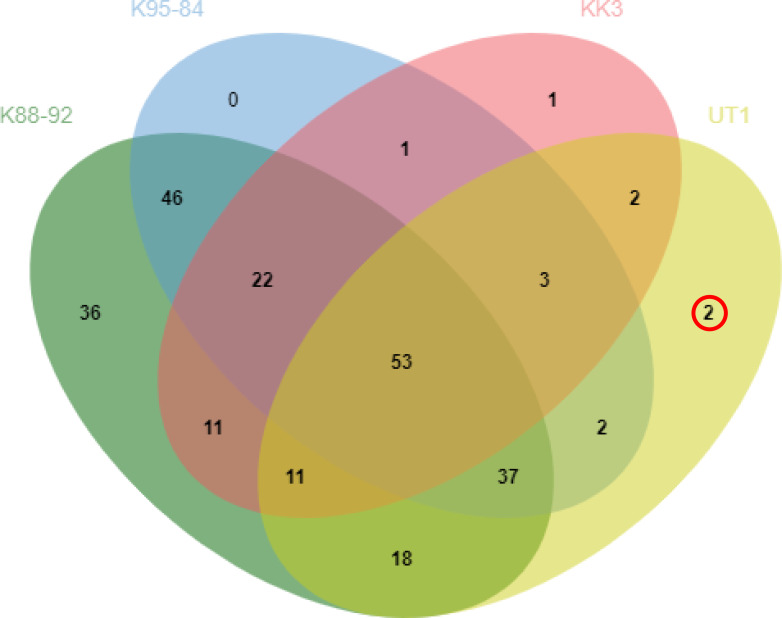
Comparison of protein expression profiles among sugarcane cultivars. The intersections of proteins identified as expressed in sugarcane cultivars infected with phytoplasma are shown for cultivars with high susceptibility to phytoplasma infection (K88-92 and K95-84), moderate susceptibility (KK3), and low susceptibility (UT1).

Previous studies have demonstrated that proteins could be used as biomarkers for monitoring distinct biochemical processes related to stress response that affect the physiological state of plants (Bechtold et al., 2009; Jangpromma et al., 2010). In the current study, we collected leaf samples from four sugarcane varieties with varying susceptibility to phytoplasma infection to identify potential candidate protein biomarkers of susceptibility. Among the 248 proteins that were identified by LC-MS/MS analysis (Table S1), the results showed different protein profiles among cultivars infected with phytoplasma. Two protein candidate biomarkers of susceptibility to phytoplasma were identified with different biological functions involved in the biochemical processes of protein folding (peptidyl-prolyl cis-trans isomerase), and metal ion binding and oxidoreductase (9-cis-eposycarotenoid dioxygenase and oxygenase) (Fig. 2; Table 2). The relationships of these proteins to phytoplasma susceptibility in sugarcane are discussed below.

The peptidyl-prolyl cis/trans isomerase (PPiase) or cyclophilin is an enzyme involved in chaperone activity, signal transduction (Schiene-Fischer & Yu, 2001; Singh et al., 2020), cell cycle control and protein secretion. PPiase is also a component of the plant immune system (Vespa et al., 2004: Dos Santos & Park, 2019), and overexpression of the peptidyl prolyl isomerase *FKBP12* gene (PaFKBP12) in *Arabidopsis* enhances resistance to abiotic and biotic stress such as high temperature, drought and salt stress ((Alavilli et al., 2018). Other examples of PPIases in host immunity reactions include Cyplophilin C-CyP isolated from Chinese cabbage (*B. campestris* ssp. *Pekinensis* L.), which inhibits growth of several fungi, including *Candida albicans*, *Rhizoctonia solani*, *Botryis cinerea*, *Trichoderma harzianum* and *T. viride* (Lee et al., 2007).

9-cis-epoxycarotenoid dioxygenase is a key enzyme in the abscisic acid (ABA) biosynthetic pathway. ABA is a crucial plant hormone that regulates plant growth, plant development and biotic/abiotic stress tolerance (Sah, Reddy & Li, 2016). The deposition of callose on the plates of sieve tubes is an early plant response to phytoplasma attack that creates a barrier to phytoplasma migration in shoots (Vitali et al., 2013). Thus, plant responses to phytoplasma infection could affect gas exchange, carbon assimilation and water transpiration (Tan & Whitlow, 2001; Endeshaw et al., 2012). When plants are attacked by abiotic stress, rapid accumulation of ABA induces stomatal closure to reduce water loss (León et al., 1996; Estrada-Melo, Reid & Jiang, 2015) and sucrose accumulation in guard cells (Lu et al., 1997). Overexpression of genes involved with ABA expression leads to increased drought tolerance as well as negative pleiotropic effects including leaf-margin chlorosis, and seed dormancy (Thompson et al., 2000).

## Conclusions

The present study reports the proteomic profiles of four sugarcane varieties with varying susceptibility to phytoplasma. Two potential protein candidate biomarkers were identified from proteins detected only in sugarcane varieties of moderate and low susceptibility to phytoplasma. These biomarker candidates constitute proteins with diverse functions (protein folding, metal ion binding and oxidoreductase), of which relationships to phytoplasma infection susceptibility are suggested. However, verification of these biomarkers using an independent method, *e.g.*, quantitative reverse-transcription PCR is needed. This is the first report of potential protein candidate biomarkers related to sugarcane white leaf disease susceptibility, which may be useful in breeding programs for selecting resistant varieties.

## Supplemental Information

10.7717/peerj.12740/supp-1 Supplemental Information 1 Supplementary Table

## Additional Information and Declarations

### Data Availability

The following information was supplied regarding data availability: The mass spectrometry proteomics data is available at ProteomeXchange: PXD028041 and JPOST: JPST001297.
